# Sex Differences in Outcomes Following Ruptured Abdominal Aortic Aneurysm Repair

**DOI:** 10.1001/jamanetworkopen.2022.11336

**Published:** 2022-05-10

**Authors:** Ben Li, Naomi Eisenberg, Miranda Witheford, Thomas F. Lindsay, Thomas L. Forbes, Graham Roche-Nagle

**Affiliations:** 1 University Health Network, Peter Munk Cardiac Centre, Division of Vascular Surgery, University of Toronto, Toronto, Ontario, Canada

## Abstract

**Importance:**

Sex differences in aortic surgery outcomes are commonly reported. However, data on ruptured abdominal aortic aneurysm (rAAA) repair outcomes in women vs men are limited.

**Objective:**

To assess differences in perioperative and long-term mortality following rAAA repair in women vs men.

**Design, Setting, and Participants:**

A multicenter, retrospective cohort study was conducted using the Vascular Quality Initiative database, which prospectively captures information on patients who undergo vascular surgery across 796 academic and community hospitals in North America. All patients who underwent endovascular or open rAAA repair between January 1, 2003, and December 31, 2019, were included. Outcomes were assessed up to January 1, 2020.

**Exposures:**

Patient sex.

**Main Outcomes and Measures:**

Demographic, clinical, and procedural characteristics were recorded, and differences between women vs men were assessed using independent *t* test and χ^2^ test. The primary outcomes were in-hospital and 8-year mortality. Associations between sex and outcomes were analyzed using univariable and multivariable logistic regression and Cox proportional hazards regression analysis.

**Results:**

A total of 1160 (21.9%) women and 4148 (78.1%) men underwent rAAA repair during the study period. There was a similar proportion of endovascular repairs in women and men (654 [56.4%] vs 2386 [57.5%]). Women were older (mean [SD] age, 75.8 [9.3] vs 71.7 [9.6] years), more likely to have chronic kidney disease (718 [61.9%] vs 2184 [52.7%]), and presented with ruptured aneurysms of smaller diameters (mean [SD] 68 [18.2] vs 78 [30.2] mm). In-hospital mortality was higher in women (34.4% vs 26.6%; odds ratio, 1.44; 95% CI, 1.25-1.66), which persisted after adjusting for demographic, clinical, and procedural characteristics (adjusted odds ratio, 1.36; 95% CI, 1.12-1.66; *P* = .002). Eight-year survival was lower in women (36.7% vs 49.5%; hazard ratio, 1.25; 95% CI, 1.04-1.50; *P* = .02), which persisted when stratified by endovascular and open repair. This survival difference existed in both the US and Canada. Variables associated with long-term mortality in women included older age and chronic kidney disease.

**Conclusions and Relevance:**

Women who underwent rAAA repair had higher perioperative and 8-year mortality rates following both endovascular and open repair compared with men. Older age and higher rates of chronic kidney disease in women were associated with higher mortality rates. These findings suggest that future studies should assess the reasons for these disparities and whether opportunities exist to improve AAA care for women.

## Key Points

**Question:**

Is there an association between sex and outcomes following ruptured abdominal aortic aneurysm (rAAA) repair?

**Findings:**

This multicenter cohort study evaluated care for 1160 women and 4148 men who underwent repair of rAAA. Women had significantly lower survival vs men up to 8 years following endovascular and open rAAA repair.

**Meaning:**

These findings suggest that further research is needed to assess reasons for this sex disparity in survival and identify whether opportunities exist to improve rAAA care for women.

## Teaser

This cohort study examines differences between men and women in survival after surgery for ruptured abdominal aortic aneurysm.

## Introduction

Sex-related differences play an important role in the natural history, diagnosis, and management of abdominal aortic aneurysm (AAA).^1^ Although the prevalence of AAA is higher in men (7.6% vs 1.3%),^2^ women experience more rapid AAA growth and a 4-fold higher risk of rupture.^3,4^ Despite the more aggressive nature of aneurysm progression in women, there are major sex disparities in AAA management.^5,6^ This difference is partly because women are underrepresented in clinical studies, limiting our understanding of the screening and treatment strategies in this population.^7,8,9^ In the event of ruptured AAA (rAAA), women are less likely to be admitted to the hospital and receive operative intervention.^10,11,12^ Overall, these factors contribute to higher AAA mortality rates among women compared with men.^13^ As a result, the Society for Vascular Surgery (SVS) recommends a lower diameter threshold for elective repair in women compared with men (5.0 vs 5.5 cm).^14^

Despite increasing efforts to improve AAA care for women, sex differences persist following aneurysm repair. Locham et al^15^ reported that female sex was independently associated with increased 30-day mortality following open (86%) and endovascular (50%) repair of non-rAAA. Other investigators have noted similar disparities in subgroup analyses of patients undergoing rAAA repair.^10,16^ In contrast, 2 studies found that sex does not significantly influence AAA repair outcomes after controlling for baseline characteristics.^17,18^ Given this conflicting evidence and the fact that most of the literature on outcomes following AAA repair exclude ruptured aneurysms or analyze data on patients with rAAA as a subgroup of a larger population containing mostly intact AAAs, further investigation is warranted.

This study assessed differences in perioperative and late mortality (up to 8 years) following endovascular and open rAAA repair in women vs men using the Vascular Quality Initiative (VQI) database. Furthermore, VQI records this data from US and Canadian centers, which provides an important opportunity to assess for regional variations in sex differences in rAAA repair outcomes.

## Methods

### Data Set

The VQI database is a set of 14 clinical registries developed by the SVS Patient Safety Organization to improve patient care.^19^ Demographic characteristics, clinical, procedural, and outcomes data from 788 US and 8 Canadian centers are submitted prospectively on consecutive patients eligible for vascular surgery, including information from their initial hospitalization and long-term follow-up.^19^ Regional quality improvement groups are accredited by the SVS Patient Safety Organization and data on academic and community physicians performing vascular procedures are reported to the VQI.^19^ Annual audits are performed to compare registry data with hospital claims to improve the reliability of submitted information.^20^

The research advisory council of the SVS Patient Safety Organization approved this study and provided anonymized data to the investigators. The need for informed consent from patients was waived by the Patient Safety Organization because the data came from a large deidentified registry.

### Patient Cohort

A retrospective cohort study was conducted using the VQI database and followed the Strengthening the Reporting of Observational Studies in Epidemiology (STROBE) reporting guideline. All patients who underwent ruptured endovascular or open infrarenal AAA repair from January 1, 2003, to December 31, 2019, were included. Patients with non-rAAA, no recorded AAA symptom status (ie, asymptomatic, symptomatic, or ruptured), or isolated iliac artery aneurysms were excluded.

### Variables

Demographic characteristics collected included age, body mass index (calculated as weight in kilograms divided by height in meters squared), which was categorized as underweight (<18.5), healthy weight (18.5-24.9), overweight (25.0-29.9), and obesity (≥30.0)^21^; primary insurer; preoperative living status; and region in which the procedure was conducted (US or Canada). Race and ethnicity were included as covariates to examine the possible association between sex and outcomes and were classified by the VQI database based on the US Census categories. The race categories included Asian, Black, White, and other (American Indian, Alaskan Native, Native Hawaiian or other Pacific Islander, more than 1 race, or unknown/unreported), and the ethnicity categories were Hispanic or non-Hispanic.^22^ Comorbidities, medications, clinical presentation, and operative characteristics were also recorded.

### Outcomes

The primary outcomes were in-hospital and 8-year all-cause mortality. Secondary outcomes were in-hospital measures: myocardial infarction, stroke, dysrhythmia, congestive heart failure exacerbation, respiratory complication, kidney failure, lower extremity ischemia, bowel ischemia, surgical site infection, and return to the operating room.

### Statistical Analysis

Baseline demographic, clinical, and operative characteristics were compared using independent *t* test for continuous variables or χ^2^ test for categorical variables. Univariable and multivariable logistic regression models were created to assess the association between sex and in-hospital outcomes, controlling for demographic, comorbidities, medications, clinical presentation, and operative characteristics. To assess time trends, procedure year was stratified into 2003-2009, 2010-2014, and 2015-2019. A χ^2^ test was performed to assess sex differences for in-hospital mortality during each time period.

All-cause mortality up to 8 years was estimated using Kaplan-Meier survival analysis, censoring patients lost to follow-up. The difference between women and men was assessed using log-rank test (unadjusted) and Cox proportional hazards regression analysis (adjusted), controlling for demographic, comorbidities, medications, clinical presentation, and operative characteristics. Stratified analysis was conducted for all repairs, endovascular repairs, and open surgical repairs. Subgroup analysis for all repairs was performed by region (US and Canada). To identify variables associated with 8-year mortality, a multivariable Cox proportional hazards regression model was created with demographic, comorbidities, medications, clinical presentation, and operative characteristics as covariates.

Our sample size calculation indicated that a cohort of 1100 patients per arm would allow for detection of a 1.2% difference in in-hospital or 8-year mortality rates assuming an SD of 10% with a type 1 error rate of 5% and power of 80%. Lo et al^17^ reported an absolute difference in 30-day mortality between women (14%) and men (2%) following open and endovascular rAAA repair.

All statistical analyses were performed using R, version 4.1.0 (R Foundation for Statistical Computing) with survival version 3.2-11^23^ and survminer version 0.4.9^24^ packages. Using 2-sided, unpaired testing, statistical significance was set at *P* < .05. Owing to the small amount of missing data for variables and outcomes of interest (<5%), complete case analysis for each covariate was performed, which is an acceptable method when the proportion of missing data is less than 5%.^25,26^

## Results

### Patient Demographics

A total of 1160 women and 4148 men underwent rAAA repair during the study period (Table 1). Compared with men, women were older (mean [SD] age, 75.8 [9.3] vs 71.7 [9.6] years); had a lower body mass index (27.7 [7.5] vs 28.3 [6.3]), with a greater proportion of women being underweight (7.7% vs 3.2%); and were more likely to be of Black race (98 [8.5%] vs 212 [5.1%]). Racial and ethnic categories with a statistically nonsignificant greater percentage of men vs women were Asian (57 [1.4%] vs 8 [0.7%]), Hispanic (122 [2.9%] vs 25 [2.2%]), White (3669 [88.5%] vs 1008 [86.9%]), and other (210 [5.1%] vs 46 [4.0%]).

**Table 1.** Demographic, Clinical, and Procedural Characteristics of Women and Men Undergoing Ruptured Abdominal Aortic Aneurysm Repair

Characteristic^a^	No. (%)		*P* value
	Women	Men	
**Demographic**			
No.	1160	4148	
Age, mean (SD), y	75.8 (9.3)	71.7 (9.6)	<.001
BMI			
Mean (SD)	27.7 (7.5)	28.3 (6.3)	.01
Underweight (<18.5)	83 (7.7)	122 (3.2)	<.001
Healthy weight (18.5-24.9)	330 (30.6)	1022 (26.5)	
Overweight (25.0-29.9)	318 (29.5)	1414 (36.7)	
Obesity (≥30)	346 (32.1)	1294 (33.6)	
Race			
Asian	8 (0.7)	57 (1.4)	.06
Black	98 (8.5)	212 (5.1)	<.001
White	1008 (86.9)	3669 (88.5)	.15
Other^b^	46 (4.0)	210 (5.1)	.12
Ethnicity			
Hispanic	25 (2.2)	122 (2.9)	.18
Primary insurer			
Medicare	637 (54.9)	1920 (46.3)	<.001
Medicaid	23 (2.0)	119 (2.9)	
Commercial	280 (24.1)	1132 (27.3)	
Military/Veterans Affairs	3 (0.3)	88 (2.1)	
Non-US insurance	19 (1.6)	93 (2.2)	
Self-pay	34 (2.9)	148 (3.6)	
Preoperative living status			
Home	1097 (94.6)	4021 (96.9)	<.001
Assisted living	54 (4.7)	73 (1.8)	
Homeless	2 (0.2)	17 (0.4)	
Region			
US	1136 (97.9)	4035 (97.3)	.21
Canada	24 (2.1)	113 (2.7)	
**Comorbidities**			
Hypertension	920 (79.3)	3182 (76.7)	.11
Diabetes	168 (14.5)	636 (15.3)	.48
Smoking			
Current	468 (40.3)	1861 (44.9)	<.001
Prior (quit ≥1 mo ago)	349 (30.1)	1445 (34.8)	
Never	322 (27.8)	736 (17.7)	
Family history of AAA in a first-degree relative	70 (6.0)	226 (5.5)	.55
Coronary artery disease^c^	204 (17.6)	958 (23.1)	<.001
Previous coronary artery bypass graft	67 (5.8)	471 (11.4)	<.001
Previous percutaneous coronary intervention	116 (10.0)	473 (11.4)	.11
Congestive heart failure	136 (11.7)	438 (10.6)	.31
Chronic obstructive pulmonary disease	412 (35.5)	1176 (28.4)	<.001
Chronic kidney disease^d^	718 (61.9)	2184 (52.7)	<.001
Dialysis	11 (0.9)	49 (1.2)	.61
Previous AAA repair	73 (6.3)	293 (7.1)	.72
Open	42 (3.6)	190 (4.6)	
Endovascular	31 (2.7)	103 (2.5)	
Previous CEA/CAS	29 (2.5)	74 (1.8)	.25
Previous peripheral artery bypass	23 (2.0)	84 (2.0)	1.0
Previous peripheral artery angioplasty/stent	41 (3.5)	129 (3.1)	.55
Previous major amputation^e^	3 (0.3)	25 (0.6)	.33
**Medications**			
Aspirin	419 (36.1)	1641 (39.6)	.04
Purinergic receptor (P2Y12) antagonist^f^	91 (7.8)	317 (7.6)	.87
Statin	470 (40.5)	1734 (41.8)	.43
β-Blocker	497 (42.8)	1757 (42.4)	.79
ACEI/ARB	287 (24.7)	1077 (26.0)	.25
Anticoagulant^g^	118 (10.2)	426 (10.3)	.84
**Clinical presentation**			
Transfer from another hospital	713 (61.5)	2504 (60.4)	.77
Lowest pre-intubation blood pressure, mean (SD), mm Hg	89.7 (33.3)	91.5 (33.3)	.11
Heart rate on arrival to operating room, mean (SD), bpm	72.7 (36.3)	74.1 (36.5)	.43
Highest intraoperative heart rate, mean (SD), bpm	88.1 (45.7)	89.1 (44.3)	.64
Mental status^h^			
Normal	820 (70.7)	3018 (72.8)	.39
Disoriented	173 (14.9)	560 (13.5)	
Unconscious	144 (12.4)	506 (12.2)	
Cardiac arrest			
No	1013 (87.3)	3647 (87.9)	.66
Yes			
Preoperatively	115 (9.9)	388 (9.4)	
Intraoperatively	16 (1.4)	60 (1.5)	
Preoperative hemoglobin, mean (SD), g/dL	10.8 (2.4)	11.7 (2.5)	<.001
Aneurysm diameter, mean (SD), mm^i^	68.0 (18.2)	78.0 (30.2)	<.001
Concomitant iliac artery aneurysm	187 (16.1)	1227 (29.6)	<.001
Operative characteristics, median (IQR)			
Time from symptom onset to incision or access, h	7.8 (4.0-20.0)	7.0 (4.0-18.8)	.56
Time from hospital admission to incision or access, h	1.5 (1.0-4.0)	1.4 (0.8-3.0)	.20
Procedure time, mean (SD), min	155.0 (105.0-225.0)	156.0 (105.0-226.0)	.21
Procedure year			
2003-2009	63 (5.4)	286 (6.9)	.18
2010-2014	391 (33.7)	1342 (32.4)	
2015-2019	706 (60.9)	2520 (60.8)	
Repair type			
Open	506 (43.6)	1762 (42.5)	.51
Endovascular	654 (56.4)	2386 (57.5)	
**Open repair**			
Exposure			.35
Transperitoneal	427 (85.6)	1518 (87.3)	
Retroperitoneal	72 (14.4)	221 (12.7)	
Proximal clamp site			
Infrarenal	241 (50.3)	905 (53.4)	.07
Above 1 renal artery	39 (8.1)	152 (9.0)	
Above both renal arteries	77 (16.1)	300 (17.7)	
Supraceliac	122 (25.5)	338 (20.0)	
Estimated blood loss, median (IQR), mL	2725 (1500-5000)	3000 (1555-5000)	.11
Total packed red blood cells transfused, mean (IQR), U	7 (3-12)	6 (2-12)	.94
Total crystalloids received, mean (IQR), mL	4000 (2725-6000)	5000 (3000-7200)	<.001
Intraoperative heparin	364 (71.9)	1312 (74.5)	.29
**Endovascular repair**			
Aortic neck			
Length, mean (SD), mm^j^	22.2 (13.8)	23.4 (11.9)	.21
Diameter; mean (SD), mm^k^	23.7 (5.3)	25.0 (5.9)	.006
Angle >60°^l^	27 (4.1)	51 (2.1)	.004
Percutaneous access	269 (41.1)	1060 (44.4)	.39
Ultrasonographic guidance for access	259 (39.6)	1021 (42.8)	.59
Aortic main device			.07
Bifurcated			.07
Infrarenal	449 (71.4)	1700 (74.2)	
Suprarenal fixation	56 (8.9)	238 (10.4)	
Aorto-uniiliac	89 (14.1)	239 (10.4)	
Bilateral aortoiliac limbs	16 (2.5)	60 (2.6)	
Aortoaortic	19 (3.0)	54 (2.4)	
Fluoroscopy time, median (IQR), min	20.6 (14.3-30.0)	20.0 (13.7-30.9)	.75
Contrast use, median (IQR), mL	100.0 (65.5-150.0)	100.0 (65.0-150.0)	.28
Estimated blood loss, median (IQR), mL	200.0 (100.0-400.0)	150.0 (75.0-400.0)	.44
Total packed red blood cells transfused, median (IQR), U	3.0 (0.0-6.0)	2.0 (0.0-7.0)	.71
Total crystalloids, median (IQR), mL	2000.0 (1250.0-3000.0)	2000.0 (1400.0-3100.0)	.09
Completion endoleak, type			
IA	24 (3.7)	44 (1.8)	.003
IB	5 (0.8)	16 (0.7)	.80
II	53 (8.1)	201 (8.4)	.79
III	20 (3.1)	38 (1.6)	.02
IV	2 (0.3)	4 (0.2)	.46
Indeterminate	28 (4.3)	68 (2.9)	.06

Abbreviations: AAA, abdominal aortic aneurysm; ACEI/ARB, angiotensin-converting enzyme inhibitor and/or angiotensin II receptor blocker; BMI, body mass index (calculated as weight in kilograms divided by height in meters squared); CAS, carotid artery stenting; CEA, carotid endarterectomy.

SI conversion factor: To convert hemoglobin to grams per liter, multiply by 10.

^a^ Complete case analysis was performed for each covariate, which removes patients with missing data for each variable/outcome of interest. This analysis was used because there was a small amount of missing data (<5%). Therefore, the denominators are not always 1160 for women and 4148 for men. The denominators reflect the total number of patients with complete data for each variable.

^b^ Other race includes American Indian, Alaskan Native, Native Hawaiian or other Pacific Islander, more than 1 race, or unknown/unreported.

^c^ Coronary artery disease was defined as a history of myocardial infarction, stable angina, or unstable angina.

^d^ Chronic kidney disease was defined as estimated glomerular filtration rate less than 60 mL/min/1.73 m^2^, calculated from recorded creatinine level using the Modification of Diet in Renal Disease equation (estimated glomerular filtration rate = 175 × (serum creatinine level in milligrams per deciliter)^-1.154^ × (age)^-0.203^ × 0.742 [if female] × 1.212 [if Black]).

^e^ Major amputation was defined as below-knee amputation or higher.

^f^ Purinergic receptor (P2Y12) antagonists included clopidogrel, prasugrel, ticlopidine, and ticagrelor.

^g^ Anticoagulants included warfarin and direct oral anticoagulants.

^h^ Mental status was defined as normal (alert and oriented), disoriented (not oriented to person, place, or time), or unconscious.

^i^ Abdominal aortic aneurysm diameter was defined as the largest anterior-posterior diameter measurement based on computed tomography, magnetic resonance imaging, or duplex ultrasonography.

^j^ Aortic neck length was defined as the measurement from the lowest renal artery to the point where the aortic neck diameter has expanded by 10%.

^k^ Aortic neck diameter was defined as the outer aortic wall diameter measured at the largest portion of the seal zone planned for device implantation.

^l^ Aortic neck angle was defined as the maximum angle between the axis of aneurysm neck and proximal portion of aneurysm sac.

### Comorbidities and Medications

A greater proportion of women had chronic kidney disease (CKD) (718 [61.9%] vs 2184 [52.7%]) and chronic obstructive pulmonary disease (412 [35.5%] vs 1176 [28.4%]), whereas men were more likely to be current smokers (1861 [44.9%] vs 468 [40.3%]) and have coronary artery disease (958 [23.1%] vs 204 [17.6%]). A greater proportion of men were using aspirin (1641 [39.6%] vs 419 [36.1%]). Other comorbidities, previous procedures, and medications were similar between groups.

### Clinical Presentation

Most patients were transferred from another hospital (women, 713 [61.5%] vs men, 2504 [60.4%]). There were no significant differences in mental status or rates of cardiac arrest between the groups. Women had a smaller mean (SD) aneurysm diameter (68.0 [18.2] vs 78.0 mm [30.2] mm), and men were more likely to have a concomitant iliac artery aneurysm (1227 [29.6%] vs 187 [16.1%]).

### Operative Characteristics

The median time from symptom onset to incision or access was 7.8 (IQR, 4.0-20.0) hours for women and 7.0 (IQR, 4.0-18.8) hours for men (*P* = .56). Time from hospital admission to incision or access was not significantly different between the groups. Repair type (endovascular vs open) was similar between sexes, with 654 (56.4%) women and 2386 (57.5%) men receiving endovascular repair.

For open repair, most exposures were transperitoneal and common proximal clamp sites were infrarenal (women, 241 [50.3%] vs men, 905 [53.4%]; *P* = .07) or supraceliac (women, 122 [25.5%] vs men, 338 [20.0%]; *P* = .07). The median estimated blood loss was 2725 (IQR, 1500-5000) mL for women and 3000 (IQR, 1555-5000) mL for men (*P* = .11). Men received significantly more crystalloids (mean, 5000 [IQR, 3000-7200] vs 4000 [IQR, 2725-6000] mL; *P* < .001); receipt of total packed red blood cells transfused was similar between men and women (mean, 7 [IQR, 3-12] vs 6 [IQR, 2-12] U; *P* = .94).

For endovascular repair, women had a smaller aortic neck diameter (mean [SD], 23.7 [5.3] vs 25.0 [5.9] mm; *P* = .006) and were more likely to have an aortic neck angle greater than 60° (27 [4.1%] vs 51 [2.1%]; *P* = .004), with no significant difference in aortic neck length between the groups. Median estimated blood loss was 200 (IQR, 100-400) mL for women and 150 mL (IQR, 75-400) for men (*P* = .44). There was no significant difference in crystalloids received or total packed red blood cells transfused between groups. Women were more likely to have a completion type IA endoleak (24 [3.7%] vs 44 [1.8%]; *P* = .003).

### Perioperative Outcomes

In-hospital mortality was higher in women compared with men (34.4% vs 26.6%; OR, 1.44; 95% CI, 1.25-1.66; adjusted odds ratio [aOR], 1.36; 95% CI, 1.12-1.66; *P* = .002). To further control for age, we analyzed a subgroup of patients aged 70 years or older and found that women continued to have a higher in-hospital mortality rate (38.4% vs 32.2%; OR, 1.31; 95% CI, 1.12-1.54; aOR, 1.30; 95% CI, 1.07-1.58; *P* = .008). Furthermore, in-hospital mortality was higher for women when stratified by endovascular repair (26.6% vs 20.9%; OR, 1.37; 95% CI, 1.12-1.67; aOR, 1.29; 95% CI, 1.03-1.63; *P* = .03) and open repair (44.5% vs 34.4%; OR, 1.52; 95% CI, 1.25-1.86; aOR, 1.53; 95% CI, 1.09-2.14; *P* = .01). Women had a lower myocardial infarction rate (4.0% vs 5.8%; OR, 0.69; 95% CI, 0.49-0.94; aOR, 0.41; 95% CI, 0.18-0.82; *P* = .02). There were no significant differences in other in-hospital outcomes (Table 2).

**Table 2.** In-hospital Outcomes Following Endovascular and Open Repair of Ruptured Abdominal Aortic Aneurysm for Women and Men

Variability	No. (%)		OR (95% CI)	Adjusted OR (95% CI)^a^	*P* value
	Women (n = 1160)	Men (n = 4148)			
Mortality	399 (34.4)	1105 (26.6)	1.44 (1.25-1.66)	1.36 (1.12-1.66)	.002
Myocardial infarction^b^	46 (4.0)	240 (5.8)	0.69 (0.49-0.94)	0.41 (0.18-0.84)	.02
Stroke^c^	31 (2.7)	95 (2.3)	1.17 (0.76-1.74)	1.43 (0.64-3.02)	.36
Dysrhythmia^d^	185 (15.9)	713 (17.2)	0.93 (0.78-1.11)	0.77 (0.53-1.09)	.14
CHF exacerbation^e^	74 (6.4)	246 (5.9)	1.10 (0.84-1.44)	1.06 (0.62-1.77)	.83
Respiratory complication^f^	284 (24.5)	1116 (26.9)	0.90 (0.77-1.05)	0.83 (0.60-1.14)	.24
Kidney failure^g^	235 (20.3)	832 (20.1)	1.01 (0.84-1.21)	0.93 (0.67-1.27)	.64
Lower extremity ischemia^h^	66 (5.7)	207 (5.0)	1.10 (0.86-1.40)	1.68 (0.97-2.87)	.06
Bowel ischemia^i^	127 (10.9)	425 (10.2)	1.00 (0.89-1.12)	1.16 (0.84-1.58)	.37
Surgical site infection	51 (4.4)	210 (5.1)	0.88 (0.64-1.19)	0.89 (0.48-1.58)	.70
Return to operating room	242 (20.9)	916 (22.1)	0.95 (0.80-1.11)	0.99 (0.69-1.42)	.96

Abbreviations: CHF, congestive heart failure; OR, odds ratio.

^a^ Adjusted for demographic characteristics (age, body mass index, race and ethnicity, primary insurer, preoperative living status, region), comorbidities (hypertension, diabetes, smoking status, family history of abdominal aortic aneurysm, coronary artery disease, previous coronary artery bypass graft, previous percutaneous coronary intervention, congestive heart failure, chronic obstructive pulmonary disease, chronic kidney disease, dialysis, previous abdominal aortic aneurysm repair, previous carotid endarterectomy/stent, prior peripheral artery bypass, previous peripheral artery angioplasty/stent, previous major amputation), medications (aspirin, purinergic receptor [P2Y12] antagonist, statin, β-blocker, angiotensin-converting enzyme inhibitor, anticoagulant), clinical presentation (transfer from another hospital, lowest preintubation blood pressure, heart rate on arrival to operating room, highest intraoperative heart rate, mental status, cardiac arrest, preoperative hemoglobin level, aneurysm diameter, concomitant iliac artery aneurysm), and operative characteristics (time from symptom onset to incision or access, time from hospital admission to incision or access, procedure time, procedure year, repair type).

^b^ Myocardial infarction was defined as a combination of clinical symptoms (ie, chest pain/dyspnea), electrocardiogram changes, and troponin level elevation.

^c^ Stroke was defined as neurological deficits persisting for more than 24 hours in the ocular, cortical, or vertebrobasilar territory.

^d^ Dysrhythmia was defined as a new cardiac rhythm disturbance requiring treatment with medications or cardioversion.

^e^ Congestive heart failure exacerbation was defined as pulmonary edema with requirement for treatment in an intensive care unit or step-down unit.

^f^ Respiratory complication was defined as pneumonia or requirement for ventilator support after initial extubation.

^g^ Kidney failure was defined as creatinine level increase greater than 0.5 mg/dL (to convert to micromoles per liter, multiply by 88.4) or requiring temporary/permanent dialysis (does not apply to patients who were receiving dialysis preoperatively).

^h^ Lower extremity ischemia was defined as a loss of previously present pulses or Doppler signals, decrease in ankle brachial index of greater than 0.15, blue toes, or tissue loss.

^i^ Bowel ischemia was defined as colonoscopic evidence of ischemia, bloody stools in a patient who died before colonoscopy or laparotomy, or clinical diagnosis by the surgeon.

**Figure 1.**
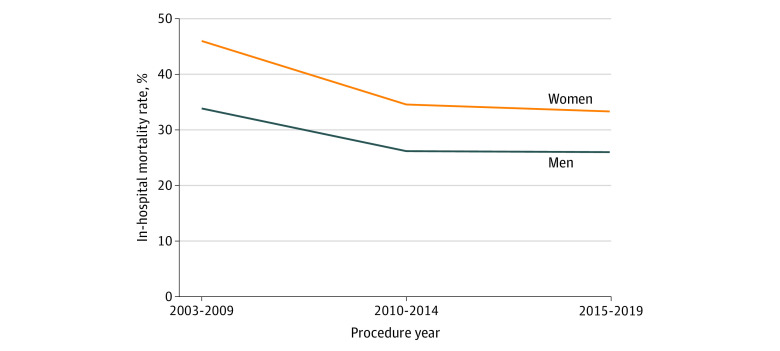
In-hospital Mortality Rate Following Endovascular and Open Ruptured Abdominal Aortic Aneurysm Repair

In-hospital mortality decreased over time for both women (2003-2009: 46.0% vs 2015-2019: 33.3%; *P* = .04) and men (2003-2009: 33.9% vs 2015-2019: 26.0%; *P* = .03). However, women had higher in-hospital mortality compared with men during each period (2003-2009: 46.0% vs 33.9%, *P* = .006; 2010-2014: 34.5% vs 26.2%, *P* = .02; 2015-2019: 33.3% vs 26.0%, *P* = .005) (Figure 1).

### Long-term Outcomes

**Figure 2.**
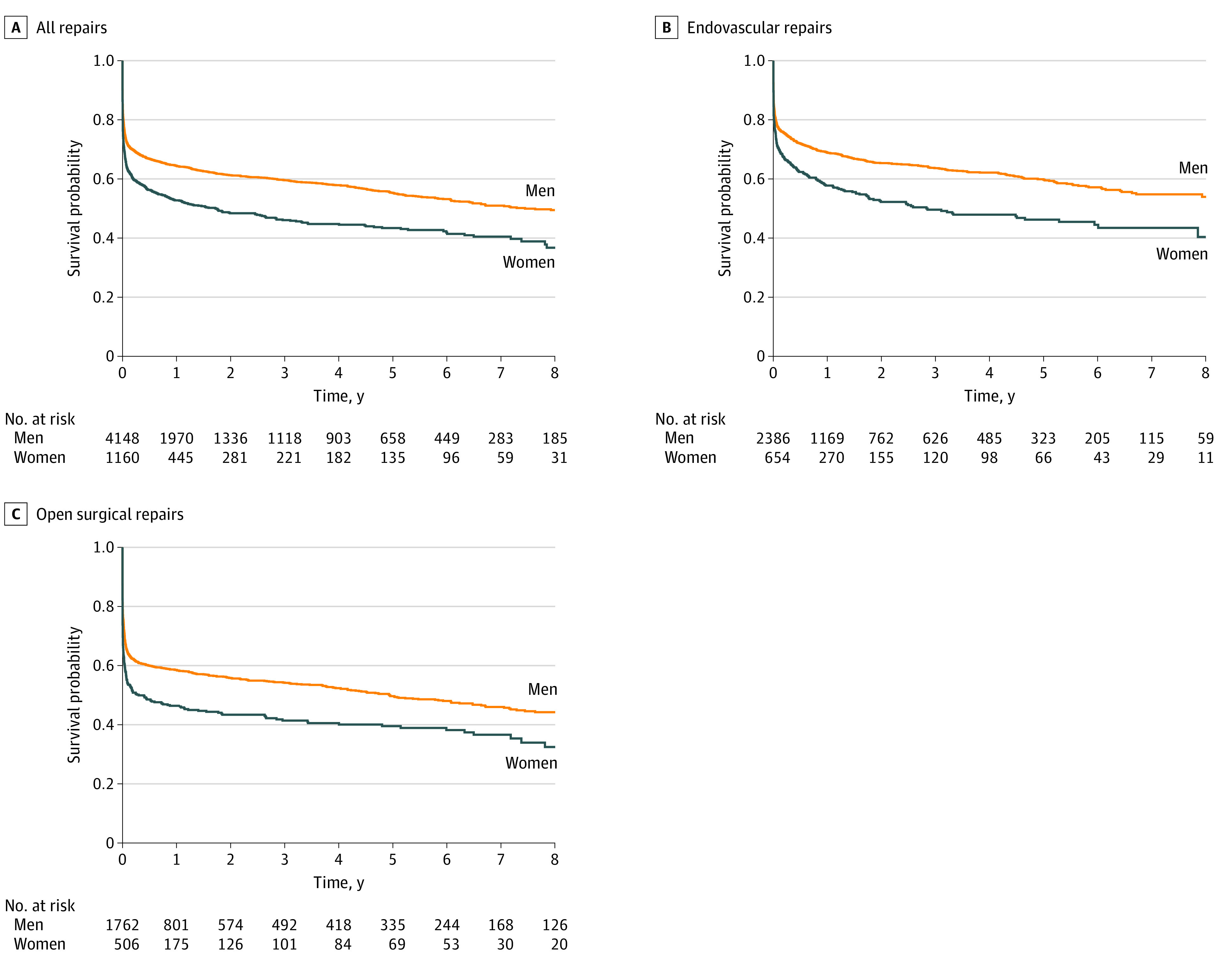
Long-term (8-year) Survival Following Ruptured Abdominal Aortic Aneurysm Repair in Women and Men Survival following all repairs (A), endovascular repairs (B), and open surgical repairs (C). Controlled for demographic characteristics (age, body mass index, race and ethnicity, primary insurer, preoperative living status), comorbidities (hypertension, diabetes, smoking status, family history of abdominal aortic aneurysm, coronary artery disease, previous coronary artery bypass graft, previous percutaneous coronary intervention, congestive heart failure, chronic obstructive pulmonary disease, chronic kidney disease, dialysis, previous abdominal aortic aneurysm repair, previous carotid endarterectomy/stent, previous peripheral artery bypass, previous peripheral artery angioplasty/stent, previous major amputation), medications (aspirin, purinergic receptor [P2Y12] antagonist, statin, β-blocker, angiotensin-converting enzyme inhibitor, anticoagulant), clinical presentation (transfer from another hospital, lowest preintubation blood pressure, heart rate on arrival to operating room, highest intraoperative heart rate, mental status, cardiac arrest, preoperative hemoglobin level, aneurysm diameter, concomitant iliac artery aneurysm), and operative characteristics (time from symptom onset to incision or access, time from hospital admission to incision or access, procedure time, procedure year).

Maximum follow-up was 16 years and median follow-up was 20.7 (IQR 9.3-58.1) months, which likely reflects the high early mortality rate. Women had lower 8-year survival (36.7% vs 49.5%; hazard ratio [HR], 1.25; 95% CI, 1.04-1.50; *P* = .02) (Figure 2A). This survival difference existed for endovascular repairs (40.3% vs 53.9%; HR, 1.25; 95% CI, 1.04-1.46; *P* = .01) (Figure 2B) and open repairs (32.5% vs 44.2%; HR, 1.47; 95% CI, 1.23-1.74; *P* < .001) (Figure 2C). For all rAAA repairs, sex differences in 8-year survival were present in both the US (HR, 1.35; 95% CI, 1.20-1.52; *P* < .001) and Canada (HR, 1.26; 95% CI, 1.03-1.98; *P* = .04). To further control for age, we analyzed a subgroup of patients aged 70 years or older and found that women continued to have lower 8-year survival (32.7% vs 39.6%; HR, 1.27; 95% CI, 1.14-1.41; *P* < .001). We performed subgroup analyses to examine whether mortality differences persist beyond the perioperative period and found that women continued to have lower 8-year survival when only those who survived beyond 30 days (57.4% vs 68.4%; HR, 1.46; 95% CI, 1.06-2.01; *P* = .02) or 90 days (61.8% vs 71.5%; HR, 1.48; 95% CI, 1.03-2.14; *P* = .03) were analyzed.

### Variables Associated With Mortality

Multivariable Cox proportional hazards regression analysis noted that variables associated with 8-year mortality included older age, CKD, preoperative dialysis, congestive heart failure, previous carotid revascularization, unconscious at presentation, cardiac arrest, and open repair. Hazard ratios are presented in Table 3.

**Table 3.** Multivariable Analysis of Covariates Associated With Mortality Up to 8 Years Following Endovascular and Open Repair of Ruptured Abdominal Aortic Aneurysm^a^

Variable	HR (95% CI)^b^	*P* value
Age	1.05 (1.04-1.06)	<.001
Preoperative chronic kidney disease	1.40 (1.18-1.67)	<.001
Cardiac arrest (preoperative or intraoperative)	1.61 (1.37-1.91)	<.001
Open repair	1.39 (1.12-1.71)	.002
Preoperative dialysis	2.82 (1.44-5.53)	.003
Previous carotid endarterectomy or stent	1.17 (1.03-1.33)	.02
Preoperative mental status (unconscious)	1.16 (1.02-1.31)	.02
Preoperative congestive heart failure	1.40 (1.08-1.81)	.01

Abbreviation: HR, hazard ratio.

^a^ Only statistically significant covariates are shown.

^b^ Covariates entered into multivariable Cox proportional hazards regression analysis: demographic characteristics (age, body mass index, race and ethnicity, primary insurer, preoperative living status, region), comorbidities (hypertension, diabetes, smoking status, family history of abdominal aortic aneurysm, coronary artery disease, previous coronary artery bypass graft, previous percutaneous coronary intervention, congestive heart failure, chronic obstructive pulmonary disease, chronic kidney disease, dialysis, previous abdominal aortic aneurysm repair, previous carotid endarterectomy/stent, previous peripheral artery bypass, previous peripheral artery angioplasty/stent, previous major amputation), medications (aspirin, purinergic receptor [P2Y12] antagonist, statin, β-blocker, angiotensin-converting enzyme inhibitor, anticoagulant), clinical presentation (transfer from another hospital, lowest preintubation blood pressure, heart rate on arrival to operating room, highest intraoperative heart rate, mental status, cardiac arrest, preoperative hemoglobin level, aneurysm diameter, concomitant iliac artery aneurysm), and operative characteristics (time from symptom onset to incision or access, time from hospital admission to incision or access, procedure time, procedure year, repair type).

## Discussion

This multicenter retrospective cohort study using prospectively collected VQI data including 1160 women and 4148 men noted several significant sex differences in rAAA repair outcomes. In-hospital mortality was higher in women (34.4% vs 26.6%), which persisted after adjusting for demographic, clinical, and procedural characteristics. This mortality difference persisted up to 8 years of follow-up (survival rate 36.7% for women vs 49.5% for men) and existed for both endovascular and open repairs. To further control for age, we noted similar findings in a subgroup analysis of patients aged 70 years or older. Furthermore, sex disparities in rAAA repair outcomes were present in both the US and Canada. Both men and women had better perioperative and long-term survival following endovascular vs open repair. Other variables associated with long-term mortality included older age and greater proportion of patients with CKD in women.

Dimick et al^27^ assessed in-hospital mortality following elective and ruptured open AAA repair using the Nationwide Inpatient Sample database. In their subgroup analysis of 2032 patients with rAAA repair, the investigators noted that women had higher in-hospital mortality. We showed similar findings in a larger study dedicated to analyzing outcomes following endovascular and open rAAA repair, further observing higher 8-year mortality.

Lo et al^17^ identified patients undergoing elective and ruptured AAA repair in the Vascular Study Group of New England database. After controlling for age, repair type, urgency, comorbidities, and other risk factors, the authors found that sex was not associated with 30-day or 1-year mortality following repair of intact and ruptured AAAs. In contrast, we noted that women had higher mortality up to 8 years following rAAA repair before and after adjusting for demographic, clinical, and procedural characteristics. Possible reasons for these differences include our study’s larger sample size for patients with rAAA (5308 vs 425 patients) and our dedicated analysis.

There are several potential reasons for the worse outcomes following rAAA repair in women compared with men. First, the natural history of AAA is more aggressive in women likely due to biological differences.^28^ Solberg et al^4^ reported that aneurysm growth rates are higher in women compared with men. Furthermore, the UK Small Aneurysm Trial noted that women have a 4-fold higher risk of rupture.^3^ Even in patients whose aneurysms were kept under surveillance, aneurysms in women are more likely to rupture at smaller diameters.^29^ This was noted in our study, with the mean ruptured aneurysm diameter 1 cm smaller in women. Second, women presented at a mean age of 4 years older than men in our study, which is important given that older age is associated with worse outcomes following AAA repair.^30^ One potential reason for the later age of presentation is less frequent screening of women owing to previous studies suggesting inadequate evidence for the benefit of AAA screening in women.^31^ For example, the US Preventive Services Task Force does not recommend AAA screening for women.^32^ However, there has been accumulating evidence demonstrating the mortality benefit from including women in screening programs.^33^ Society for Vascular Surgery guidelines suggest a 1-time AAA screening ultrasonogram in men and women aged 65 to 75 years with a history of tobacco use (grade 1A).^14^ The implementation of these screening guidelines may reduce the rates of older presentation in women and improve postoperative outcomes. Third, women were more likely to present with CKD, which was associated with higher mortality. Previous studies have noted that women have a higher prevalence of CKD but are disadvantaged by reduced access to deceased donor transplantation.^34^ Sex disparities in kidney care may contribute to worse outcomes in women, given that CKD is associated with substantial perioperative mortality and morbidity.^35^ Fourth, women are more likely to have unfavorable anatomy for endovascular repair, as demonstrated by the higher rates of aortic neck angulation greater than 60° in our cohort. This finding is corroborated by previous reports.^15,17^ In our study, the greater aortic neck angulation in women likely contributed to higher rates of completion type 1A endoleak, which is associated with higher mortality risk.^36^ Furthermore, there was a statistically nonsignificant greater proportion of women requiring a supraceliac clamp (25.5% vs 20.0%; *P* = .07), which may have contributed to worse outcomes following open repair.^37^ Fifth, the underdiagnosis and undertreatment of AAA in women has been previously documented.^38,39^ Unconscious and conscious sex bias and discrimination prevalent in medicine and society may extend to AAA management.^40^ Opportunities exist for clinicians to improve gender equity, including participation in women’s health education, implicit bias training, and cultural change.^41,42,43^

Another important finding in our study was that patients undergoing endovascular repair had a higher 8-year survival rate compared with those undergoing open repair. Several clinical trials have reported the feasibility of endovascular rAAA repair by showing similar perioperative outcomes compared with open surgery.^44,45,46,47^ Observational studies have suggested improved perioperative mortality for endovascular repair.^48,49,50^ Our 8-year analysis is, to our knowledge, among the first to report the possible long-term mortality benefit of endovascular over open rAAA repair. In our study, estimated blood loss was greater than 2500 mL for open repair compared with 200 mL or less for endovascular repair. This finding of increased physiologic stress associated with open repair is corroborated by others^48,49,51^ and supports the SVS recommendation to proceed with endovascular over open repair for the treatment of rAAA in patients with suitable anatomical characteristics.^14^

### Limitations

This study has limitations. First, this was a retrospective analysis of a large, prospectively maintained registry with potential for coding errors. To reduce these errors, the VQI conducts annual audits of clinical data reported by hospitals.^52^ Second, the VQI database only captures patients who undergo interventions and data were not available for those with rAAA who did not receive repair. Third, there were significantly more US patients than Canadian patients included in our study. This is likely because the US population is approximately 10 times larger than the Canadian population.^53,54^ Fourth, our analysis of factors associated with mortality following rAAA repair was exploratory. Future studies dedicated to understanding causes of sex differences in rAAA repair outcomes may be useful.

## Conclusions

This study noted important sex differences in the patient profile, procedural characteristics, and outcomes following endovascular and open rAAA repair. Women were older, more likely to have CKD, experience rupture at smaller aneurysm sizes, and present with unfavorable aortic neck angulation. Compared with men, women had higher perioperative and late mortality rates up to 8 years following rAAA repair. Both men and women had better survival if they underwent endovascular rather than open repair. Important variables associated with higher mortality in women included older age and CKD. Future studies would be useful to assess the reasons for these disparities and whether opportunities exist to improve AAA care for women, including more frequent screening, better rupture risk stratification, and strategies to reduce gender bias and discrimination.
